# The search for the principle of justice for infertile couples: characterization of the brazilian population and bioethical discussion

**DOI:** 10.1186/s12910-023-00947-4

**Published:** 2023-09-04

**Authors:** Drauzio Oppenheimer, Francisca Rego, Rui Nunes

**Affiliations:** 1 Faculdade de Medicina de Itajubá, Av. Rennó Junior, 368, São Vicente, Itajubá, CEP 37502-138 MG Brasil; 2 Faculdade de Medicina da Universidade do Porto, Porto, Portugal

**Keywords:** Assisted reproduction treatment, Bioethics, Infertility, Justice, Principle of justice

## Abstract

**Background:**

Infertility is an increasingly prevalent disease in society and is considered by the World Health Organization to be a public health problem. An important ethical issue arises from the clarification of reproductive rights in a fair and equal way. The objective of this study was to deepen and update the knowledge and discussion about the difficulty of accessing infertility treatments in Brazil.

**Methods:**

A cross-sectional observational study was carried out through the application of an online questionnaire that collected the socioeconomic characteristics of couples and identify how barriers to infertility care affect the most vulnerable populations. We included couples who sought medical assistance to achieve pregnancy at two clinics in the states of São Paulo and Minas Gerais.

**Results:**

A total of 201 questionnaires were analyzed. Most couples self-declared as white and the average age of wives was 36 years and husbands 38 years. 65% (65%) of couples would proceed with the treatment in a different city to which they lived, 37% evaluated as having easy access to a medical specialist only after indication, and more than half of the participating have thought about giving up the treatment due to some difficulty in accessing it. 39% of participants sought more than one medical service to find better reception, 42% of couples sought more than one medical service to define where it would be better financially, and 67.2% referred to the high cost of treatments, that is, financial issues, as a great difficulty in accessing medical services and/or treatment. Although 72.6% of couples considered having a good quality of life, 54.2% admitted that infertility and the search for treatment generated anxiety/stress in the couple’s life.

**Conclusion:**

There is a need for public education on reproductive health and for policymakers to raise awareness of the importance of the difficulty that many couples face in seeking treatment to become pregnant, especially in countries with less financial resources. Indeed, it is commonly accepted that there is a universal human right to access healthcare of appropriate quality as a matter of justice. Discussion of access to reproductive technologies should be considered taking into account the longstanding ethical debate regarding fertility, fecundity, and infertility, as well as reproductive care.

**Supplementary Information:**

The online version contains supplementary material available at 10.1186/s12910-023-00947-4.

## Background

Infertility, defined as the inability of a couple to conceive after one year of regular sexual intercourse and without the use of contraceptive methods [1], can occur due to several factors such as alterations to the male or female reproductive tract or its endocrine control, patient age, and metabolic diseases, among other causes. Infertility directly affects the physical and psychological well-being of patients [2]. It is also noteworthy that infertility has become a significant public health and political problem in the last 25 years [3].

For Mascarenhas et al. (2012), estimates of the worldwide prevalence of infertility indicate that approximately 70 million couples will require medical assistance to achieve pregnancy [4]. Several studies show that infertility is an increasingly prevalent disease in society and, therefore, there is a movement to offer treatment to everyone [5, 6]. Although it is still currently necessary to discuss and reinforce reproductive rights, in 1994, during the historic International Conference on Population and Development (ICPD), held in Cairo, the prevention and treatment of infertility were recognized as basic components of rights and care to sexual and reproductive health [7, 8].

Furthermore, in 2001, the World Health Organization (WHO) organized a meeting entitled “Medical, ethical and social aspects of assisted reproduction”. At that meeting, in addition to the WHO recommending that infertility be considered a global public health problem, it also recognized it as a disease [9], with a recommendation that infertility management be added to national health education services. It was also recognized that there is a need for affordable assisted reproduction treatments in countries with few resources, using simplified protocols [5].

It is important to admit that infertility and its treatments are the focus of ethical questions about the implications of its use and its accessibility, involving individual, social, political, legal, economic, religious, and gender issues, thus bringing important debates within the scope of bioethics. In other words, a very important ethical issue is envisaged: the clarification of reproductive rights in a fair and egalitarian way [10]. Therefore, while on one hand it is the responsibility of the State, as a representation of society, to provide and promote health and the well-being of the population, on the other there are many ethical discussions regarding what are the limits of this responsibility, especially considering the financial burden of treatments (both to individuals and to the State), limited resources that lead to election of priorities, novel familiar organizations, and even quality of life of the children and families in vulnerable populations [11].

The inequality gap between having or not having access has been increasing yearly. Studies show that, in the United States, for example, most patients who treat infertility through assisted reproduction protocols are white, have a high educational level and high financial income, and also have access to special health insurance, which includes this benefit. In an analysis of family growth research, it was found that women with infertility who did not have access to evaluation and treatment were the ones with the lowest family income and level of education and without health insurance [12].

It is relevant to point out that, in Brazil, the right to family planning is provided for in the Federal Constitution of 1988 [13], however, a large part of the population remains without access to assisted human reproduction services. Low-income persons with infertility face difficulties in seeking free care or are unable to have this right. Another important factor, according to Martins et al. (2019) is that, in addition to the difficulty of access, many couples are unable to solve the problem of infertility due to the lack of information, with negative consequences in the family and social environment [2].

According to Inhorn and Patrizio (2015), only 51% of patients with infertility, in countries with few resources, seek medical care, precisely because the service is expensive, and difficult to access, without the possibility of absence from work and travel costs [14]. In recent years there has been an increase in the number of human reproduction clinics around the world, but they are still concentrated in large centers and are accessible to higher-income couples, as mentioned by the European Society of Human Reproduction and Embryology (ESHRE) task force, they are islands of high-tech assisted reproductive treatment in a sea of poverty and medical malpractice [15, 16]. Few couples have equitable and fair access to these services. The financial costs reflect catastrophically on the family budget (exceeding 40% of the family budget) threatening family survival. In this context, they resort to personal loans, take on more work positions, limit themselves to buying food and clothes, and exhaust their savings [14].

In addition to facing difficulties in achieving fatherhood and/or motherhood, the psychosocial consequences of infertility in high-income countries lead to depression, anxiety, loss of self-esteem, relationship difficulties, lower quality of life, and isolation. In low-income countries, especially in some religious beliefs, having children is a family commitment and there is a stigmatization of women, often considered “at fault” for infertility, leading to domestic and psychological violence, marriage dissolution, fear of the husband separating for another wife, disinheritance, and social isolation. This can result in physical and psychological abuse, polygamy, and even suicide [17].

The principle of justice guarantees, or should guarantee, access in an equitable way and can be translated as the equitable, fair, and universal distribution of health-related benefits. In Brazil, the practical application of this principle must allow access to all people, to the use of assisted reproduction techniques, because, health is everyone individual’s right in addition to being a duty of the State [13]. Thus, on ethical issues, two important points stand out: barriers to access to reproductive care and infertility as a global public health problem [18].

Finally, scientific advances in the medical field and new technologies of assisted human reproduction provide many infertile couples with the realization of the dream of family formation, but there is an increasing incidence of infertility, especially in developing countries, and even more observed where there is socioeconomic inequality, excluding treatment [19]. Thus, the present study aims to address the difficulty of infertile couples to access infertility treatments in developing countries, focusing on the essence of the principle of justice.

## Objectives

The present study aimed to deepen and update the knowledge and discussion on the difficulty of accessing infertility treatments in developing countries, focusing on the principle of justice.

The following specific objectives were determined:

- Identify the socioeconomic characteristics of couples with infertility assisted in the states of São Paulo and Minas Gerais - Brazil;
- Identify how barriers to infertility care affect the most vulnerable populations.

## Methods

A cross-sectional, multicenter observational study carried out through a bibliographic survey and application of an online questionnaire (Google Forms), developed for the purpose of this study, to couples who sought medical aid to achieve pregnancy at the Oppenheimer Clinic in Santa Rita do Sapucaí (MG) and the Clinic of Fertility and Human Reproduction Gera (SP) between September 2021 and January 2022. An English translation is provided as supplementary material to this manuscript (supplementary file 1). This study was approved by the Research Ethics Committee (CEP) of the Itajubá School of Medicine (CAAE: 50434521.6.0000.5559). The study consisted of 211 couples, who responded appropriately to the questionnaire. All included participants signed an informed consent form agreeing to participate in the study, and Brazilian National Research Ethics Council guidelines were followed.

The questionnaire was applied to couples over 18 years of age and participants who did not correctly fill out the questionnaire were excluded. Because the study focused on access to care for couples who had been actively trying to conceive without medical assistance previously, homosexual couples and single women were also excluded from this analysis.

### Data collection

The bibliographic survey was carried out in PubMed, Google Scholar, LILACS, Web of Science, and SciELO databases and legal decrees. For the research, a narrative review of literature was carried out including all articles containing the keywords: infertility, bioethics, ethics based on principles, justice, and assisted reproduction treatment published in English from 1998 to the present date.

### Preparation and validation of the questionnaire

The two following stages after the literature review were questionnaire elaboration and pilot study validation, as described below.

#### Elaboration of the questionnaire

The production of the questionnaire items was carried out by researchers in the area of human reproduction and bioethics. Thirty questions were prepared, 24 of which were nominal (yes/no) and 06 were open-ended, with the aim of absorbing knowledge about personal, cultural, economic, and social identity, in addition to identifying the main difficulties of participants in accessing reproductive care. The questions were organized according to the subjects: (a) sociodemographic characteristics of the couple, seeking to know the age, color/race, and level of education of the participants; (b) time of infertility, attempts to become pregnant and cause of infertility; (c) impact of infertility on the couple’s life, that is, whether the couple’s relationship was strengthened or weakened due to infertility and if they are going through or have gone through moments of anxiety and stress, in addition to pointing out the perception of quality of life; (d) experiences of seeking care and treatment for infertility, identifying the city where the couples underwent or will undergo the treatment and the difficulties in accessing the necessary treatment, and finally (e) social and economic conditions.

The questionnaire was validated through the elaboration of a scientific article already submitted for publication.

#### Pilot study

For the pilot study, the online questionnaire was applied to 15 couples over 18 years of age who sought medical treatment for marital infertility and agreed to participate in the study. It was possible to observe whether the participants found it difficult or not to answer the questionnaire and, after statistical analysis, whether the information obtained was satisfactory.

### Questionnaire application

After validation in the pilot study, the questionnaire was applied to 211 consecutive couples attending the Oppenheimer clinic between September and December of 2021.

### Statistical analysis

For statistical analysis, SPSS 18.0 software for Windows was used. Initially, a descriptive analysis of the data was performed. For continuous numerical variables, mean, standard deviation and 95% confidence interval (95% CI) of the means were presented. For discrete numerical variables, median, interquartile range, minimum and maximum were presented. For frequencies, the percentage and its 95% CI were calculated. Correlation between the variables was verified using Pearson’s correlation test (between two continuous numerical variables) or Spearman’s correlation test (all other cases). An alpha of 5% was adopted.

## Results

The questionnaire was answered by 211 couples who sought medical assistance to achieve pregnancy (104 couples from the Oppenheimer Clinic in Santa Rita do Sapucaí – MG and 107 couples from the Clinic of Fertility and Human Reproduction Gera – SP). As they were not considered cases of infertility, homosexual couples (6 questionnaires) and couples where the partner indicated having had a vasectomy (4 questionnaires) were excluded. In this way, a total of 201 questionnaires were analyzed and the results are presented according to the subjects indicated above.

### Sociodemographic characteristics of the couple, time of infertility, attempts to become pregnant, and level of education

Starting with the analysis of the age of the partners, it was identified that the average age of the wives was 36.7 years and the average age of the husbands was 38.6 years, observing couples that were still young. The duration of infertility ranged from 6 months to 25 years, with a mean time of 6 years.

Regarding the number of treatments performed to get pregnant, the analysis showed that most couples were making the first or second attempt (Table 1), however, it is important to report that 5.47% of couples showed a lot of distress in the questionnaire when answering that they no longer knew, because they are various, the exact number of attempts.

**Table 1:** Individual characteristics of respondents to the questionnaire

Variable	Value
Wife age (years)	
Mean (Standard Deviation)	36.7 (5.30)
95% CI	[35.9; 37.4]
Min – Max	20–50
Husband age (years)	
Mean (Standard Deviation)	38.6 (6.96)
95% CI	[37.7; 39.6]
Min – Max	20–63
Infertility (years)	
Mean (Standard Deviation)	6.0 (4.29)
95% CI	[5.4; 6.6]
Min – Max	0.5–25
Number of treatments (attempts)	
Mean (Standard Deviation)	2.0 (2.05)
95% CI	[1.7; 2.2]
Min – Max	0–12

The questionnaire exposed the level of education for both partners of the couple. It was observed that, among the wives (first partner), 19% indicated that they had attended high school, 32% had completed higher education and 38% had postgraduate degrees. Among the husbands (second partner), 36% had an education up to high school, 27% had completed higher education and 20% had a graduate degree.

When analyzing the answers referring to color or race, it was found that most participants (69.2% - wives and 72.6% - husbands) declared themselves white, followed by brown (22% - wives and 20.4% % - husbands) and black (6.5% - wives and 5.5% husbands). Asian races (1.5% - wives and 1.0% - husbands) and indigenous races (1.0% - wives and 0.5% - husbands) were the least indicated options.

In order to characterize the couples, the questionnaire sought to understand whether the participants already had children and whether they knew the cause of infertility. Only 18% of couples already had a previous conception. And, it was shown that in 47.3% of couples, the cause of infertility is female, in 16.4% the cause is male, in 18.4% the cause is considered mixed and in 18% the infertility is without apparent cause.

### Experiences of seeking care and treatment for infertility and difficulties in accessing treatment

Interestingly, when asked about the city in which they lived and the city where they would undergo the treatment to achieve pregnancy, 65% of the participants indicated that they would proceed with the treatment in a different city in which they lived, indicating how Brazilian couples search for treatment. Following this reasoning, it was observed that 34.3% of the couples considered having easy access to an assisted reproduction clinic and to infertility treatment options only after searching in a different city in which they lived, and, still, 21.4% indicated difficult access.

The questionnaire showed that 39% of couples considered it easy to access a specialist and medical services to treat infertility, 37% rated it easy to access only after an indication or help, and 24.4% found it difficult to access a specialist doctor. It is important to highlight that more than half of the participating couples (51.7%) have already thought about giving up on treatment due to some difficulty in accessing them.

Although there are adversities, 61.2% of couples felt welcomed in the search for medical services and treatment for infertility, but it still cannot be overlooked that 28% of couples felt welcomed after some frustrating experiences, and 11% were not welcomed. To understand what couples prioritize when choosing a specialized medical service, we asked whether the couple sought more than one service and what motivated them. The answers showed that 39% of the participants sought more than one medical service until they found better care, 42% of the couples sought more than one medical service until they defined where it would be better financially, 14% answered that they did not seek more than one medical service due to lack of services in their location and only 5.5% did not seek more than one service due to lack of knowledge. Information was also obtained that 77.6% of couples are not aware of free treatment in other countries.

As already indicated in the previous items of the questionnaire, 67.2% of couples demonstrated the high cost of treatments, that is, financial issues, as the greatest difficulty in accessing medical services and/or treatment and 13% of couples pointed to both financial and location difficulties as factors that made access difficult. In this group of participants, only 0.5% of couples think that the greatest difficulty in access involves discrimination.

### Impact of infertility on the couple’s life and perception of quality of life

Some items of the questionnaire addressed quality of life and personal sentiments experienced by couples. Regarding the impact of infertility on the couple’s life, 40% said that the relationship was strengthened by suffering infertility, 21.4% of the couples indicated that the relationship was weakened and 39% did not feel a positive or negative impact on the relationship. Then, 54.2% of couples admitted that infertility and the search for treatment generated anxiety/stress in the couple’s life from the beginning, and 33% indicated these feelings only after some treatment attempts. However, despite the moments of anxiety and stress, 24.4% of the participants consider having a great quality of life, 72.6% consider having a good quality of life and only 3.0% of couples consider having a bad quality of life.

### Social and economic conditions

We also sought to deepen our knowledge of the socioeconomic status and family income of the participating couples. For 78.1% of couples, the cost of treatment impacted the family budget and 48.3% of couples had to resort to loans or have assets to be able to afford treatment. In addition, 65.2% of the couples found it necessary, after diagnosis and treatment, to wait to obtain financial resources to perform the procedure and 45.3% sought coverage by health plans or the public health system, but they remained helpless.

It was observed that most participating couples have a family income of up to 10,000 reais (1 real – 0,18 €). The option of income between 3,000 and 5,000 was selected by 45% of couples, 31.3% of participants indicated income between 5,000 and 10,000, 8.5% of couples said they had a family income between 10,000 and 15,000, 6.5% of couples have an income between 15,000 and 20,000, only 2% of couples reported having an income of 25,000 or more, and 7% of couples selected an income range not indicated in the options offered, suggesting a family income below 3,000 reais.

Additionally, couples with a higher income range had better overall self-assessed quality of life (p = 0.010). Furthermore, it was noted that having better financial conditions is associated with easier access to the assisted reproduction clinic and treatments (p˂0.0001) and these couples think less about giving up on treatment due to some difficulty in accessing them. (p = 0.021).

It is important to note that the questionnaire left free space for the participating couples to feel free to enter comments. Most of the comments (34.72%) were to indicate the immense financial difficulty to access and carry out the treatments suggested by the medical service. Additionally, couples put into words their anxiety about achieving the dream of motherhood/fatherhood and how distressing the whole process can be. For example, some comments indicate: anxiety was an impacting factor among treatment attempts; infertility generates feelings of anguish and frustration; the way is long and painful; the treatment generates many frustrated expectations; my dream is to be a mother, but the treatment is distressing.

Finally, the questionnaire presented the information that several participating couples (71.1%) have friends or acquaintances who also seek treatment to achieve pregnancy and that 27.4% of these friends or acquaintances managed to access treatment with difficulty, and that 11% are still helpless, that is, they have not yet been able to access treatment.

## Discussion

Infertility, according to the WHO, is a public health problem and affects about 186 million people worldwide. In Brazil, an estimated 8 million individuals may bear this condition [20, 21]. As a treatment, assisted reproduction techniques allow millions of couples who experience infertility the possibility of becoming pregnant, especially in low- and middle-income countries, where we find a higher incidence of infertility [4–6, 22].

The results observed in this study allowed us to understand that the narrative of couples who seek pregnancy through assisted reproduction techniques presents important reflections on the experience of undergoing infertility and, also, reflections on the relationship between the difficulties access to treatments and the socioeconomic characteristics of these couples. The reality of working as an infertility specialist in Brazil, working in the Minas Gerais state countryside, is in line with the responses of the participating couples: a large number of anguished patients are received without a correct investigation of the reason for infertility, and many couples never considered they would be able to commute to larger cities, due to their socioeconomic and cultural level, to carry out this investigation with a specialist. Unfortunately, a limitation was observed in the study by applying a questionnaire because there was no evaluation by a committee of experts before the pilot test.

Indeed, in most countries access to healthcare of appropriate quality is considered a basic and fundamental human right, even a universal human right [23]. It follows that all societies, more or less developed, should promote an intensive debate on the establishment of priorities in healthcare so that it is socially determined, in accordance with the principle of public accountability, if infertility treatments should be offered in the basic healthcare package delivered to all citizens [24]. This is particularly challenging given the many financial burdens already imposed by increased cost of healthcare (both in developed and in developing nations), as well as novel constitutions of familial relations that render infertility treatment necessary for patients that would not fall under the classic definition of infertility (i.e. after one year of unprotected sexual relations) [11]. Even in countries where infertility treatment is gratuitously offered to infertile patients, there are barriers imposed, such as female age, number of previous children, or number of attempts [25]. It is of utmost importance, then, that such discussions are carried out involving lawmakers, health providers, stakeholders in the access-to-care pipeline and, especially, patients themselves – especially considering that medical treatment and assisted reproduction are quite often not the only options for managing infertility [25].

After overcoming the access barriers, at first, with adequate care and a confirmed diagnosis, the couples demonstrate satisfaction and are extremely happy, believing that the infertility problem can be solved and, thus, the anguish and anxiety will end. However, from the moment of indication of in vitro fertilization, the costs of the in vitro fertilization treatment (since it is in the private sector), and the need to perform it in another center, a large percentage gives up on having the procedure performed, while other couples responded to be still trying to find financial means for treatment. Only a fraction with higher socioeconomic conditions declared to be able to carry out the treatment immediately.

Corroborating a study by Alon et al. (2021), it was observed that access to assisted reproduction techniques has a cultural inequality, where couples with more schooling (university and graduate studies) were those who managed to resort to specialized services, with 70% of the wives and 47% of the husbands having a higher level of education [26]. A survey by Iba et al. (2021) demonstrated that there is an association between schooling and the search for medical help, exemplifying that people with higher education exhibit more behavior of seeking medical help; in addition, couples with better financial, psychological, social and cultural conditions also present greater search for treatment. Therefore, this group has an awareness of infertility problems, leading to coping with the problem and seeking treatment [27].

Another important finding of the results of the current study is that a large percentage of the wives have a high level of education. Chambers et al. (2013) emphasize that the high level of schooling of wives occurs by postponing pregnancy by concentrating on the profession, and this leads to differences in the pattern of infertility and procreation due to greater purchasing power and facilities in performing in vitro fertilization [28].

As observed, a large percentage of the patients who answered the questionnaire identified themselves as white, showing the racial disparity in access to assisted reproduction treatments [29, 30]. According to Butts (2021), black patients begin the search for infertility research 6 to 15 months after white patients do, and they still take a longer time to perform adequate treatment, which negatively reflects on ovarian reserve, leading them to undergo more cycles of treatment with greater costs and lesser successes, characterizing racial inequalities and the absence of equal access to treatments [31].

The result of the study also showed that all care for couples who seek to solve infertility problems in Brazil is carried out in private clinics, directing the treatment to only a portion of the population with greater purchasing power, excluding or creating difficulties for less economically favored couples. The main barrier to accessing treatments suggested by the medical service, according to the study, is financial. Hammarberg and Kirkman (2013) agree and state that the search for financial resources impacts the family budget and possessions, with loans or withdrawal of treatment [16], highlighting the need to make assisted reproduction treatments more accessible through public offerings or supplementary health plans, in pursuit of the principle of justice [32, 33].

Another point, demonstrated in the study and also impacting treatment withdrawal, is the difficulty of accessing specialists in the region where they live, limiting specialists and clinics to large centers and many take years for specialized care. This disparity in access to infertility care due to socioeconomic conditions, ethnicity, and the geographic area does not guarantee equity, and promoting health is guaranteeing rights and intervening in inequalities in the distribution of goods and services [34, 35].

Additionally, infertility is known to cause psychological and social distress such as fear, guilt, depression, marital stress, emotional abuse, intimate partner violence, divorce and partner abandonment, social isolation, economic deprivation, loss of social status, and in some regions (e.g., Africa and Asia), even famine, disease, violence-induced suicide, and loss of dignity in death [16, 17, 36]. To identify this impact on patients’ lives, the questionnaire also addressed questions about the perception of the quality of life of infertile couples, identifying moments of anxiety, anguish, and stress due to infertility and the search for treatment. It was noted that couples manage to maintain hope, but emphasize moments of suffering and pain. In addition to showing stress throughout the process, from getting access to a specialist to completing the treatment, the couples stated that the path to realizing their dream of motherhood/fatherhood was very distressing.

After characterizing the barriers and pointing out the disparities in access to infertility treatments, it became possible to deepen the bioethical argument, in particular, the principle of justice. Questions arise regarding the health of the population and the health system, including its structure, financing, and management of its resources in relation to public policies [37]. In Brazil, the 1988 Constitution establishes health as a social right, but in the current scenario and in the political culture of government practices, the State lacks commitment to universal access to this right [22, 33]. Due to this, intervention bioethics seeks to demonstrate the importance of investments and government actions that prioritize healthcare for the less favored classes [32]. Bioethics can contribute to the search for improvements in health and justice conditions [38, 39], and this search must be broad, that is, involve social conditions, discrimination, difficulty in accessing information, and to the treatments proposed by the healthcare system.

Thus, according to some researchers, there are three ethical principles that provide an ethical basis for assisted reproduction treatments: the principle of freedom, the principle of utility, and the principle of justice [10, 40], which refers to equal treatment and fair distribution of State funds for health, research, and prevention. However, medical ethics is based on society’s moral, religious, and philosophical ideas and principles and is influenced by economics, policies, and laws [41]. This creates tension between the principles of justice and utility, which can result in a disparity in the availability and access to assisted reproductive technology services between the rich and poor [42, 43].

It is pertinent to suggest that actions should be implemented to achieve reproductive justice around the world and especially in countries with fewer financial resources. This includes promoting educational programs to prevent infertility before it sets in, early detection of diseases linked to infertility, such as sexually transmitted infections and post-abortion consequences, and reversing global obesity, smoking, and alcohol with its reflection on the hormonal axis of patients [16]. Furthermore, a greater ethical and sociocultural awareness of infertility and the impact of childlessness is warranted, where fatherhood and motherhood are socially obligatory, seeking to support these patients who live in marginality and ostracism, proposing new paths such as adoption or even support for not having children, deconstructing old concepts. Finally, it is of especial importance to simplify diagnostic procedures and infertility treatment, bringing lower cost protocols for ovarian stimulation and IVF procedure, being available and within reach of a larger part of the population. A potential solution is to promote actions that have a public-private partnership with political and governmental support [16].

The Brazilian law 11.935/2009 of mandatory coverage regarding family planning does not state the types of treatment that should be covered. Thus, it is understood that the exclusion of coverage for treatments involving infertility is subject to legal questioning. There was a modification of this law from its original wording in Art. 35-C, by including in the list of mandatory coverage of health plan procedures related to family planning [44]. Even in the face of some judicialization, the judiciary has been more favorable to the derogation of item III of Art. 10 of the Health Insurance Law. Therefore, it is necessary to prioritize public health initiatives to prevent infertility, interventions to reduce costs, and provide specific treatments [5, 18].

Affordable infertility treatment can only be successfully introduced in developing countries if sociocultural and economic prerequisites are met and governments can be persuaded to support its introduction. Establishing contacts with the authorities and discussing infertility in public health structures is fundamental [12, 36, 37]. It also raises the question of contributions from the pharmaceutical and medical industry, with cheaper equipment, lower cost medication, the manufacture of basic products and laboratories with lower costs, and simplification of procedures so that are accessible, of good quality, and of low economic cost [12, 17].

Global access to infertility care in developing countries can only be achieved when it is linked to family planning programs, and national and international health strategies. This would entail a shift in the conventional view, which has focused on reducing total fertility rates, to also focus on infertility in patients at need. This would thus emphasize that infertility prevention measures, especially in developing and low-income countries, are essential and must be effective and reach the most vulnerable populations. It is suggested that, in Brazil, these measures are part of a government program within the Sistema Único de Saúde - SUS (Health Single System) [21, 36, 45].

Finally, since infertility is a disease and is considered by the WHO as a public health problem, ways must be sought for its prevention and treatment, seeking egalitarian health. Therefore, the principles of bioethics are fundamental and should guide the dilemmas faced. The principle of justice establishes a fundamental condition of equity and impartiality, and everyone must be treated ethically and given what is due to them.

## Conclusions

Infertility is considered by WHO to be a public health problem with psychological and social repercussions. Its incidence has increased, mainly in regions and countries with less favored socioeconomic and cultural conditions, precisely where access to treatment resources is non-existent. The main barrier to accessing treatment is financial, as the costs are high and have a negative impact on the family budget or even make it impossible to do so. Also, treatments are performed only in private clinics in large centers and patients are forced to move to these regions or give up treatment.

In this study, we tried to find out the conditions of access to infertility treatments in a justice and equity approach. Methodologically this study enrolled only heterosexual couples with clinical diagnosis of infertility. Single women or homosexual couples were excluded because access to reproductive technologies in those cases has a different ethical context. Nevertheless, future studies should also approach this issue. Bioethics, in its critical and interventional role, with a focus on protecting the more fragile members of society, should contribute in the discussion and consolidation of reproductive justice.

The pandemic limited the study because many patients abandoned or postponed treatment during this period. Other studies must be carried out, given the relevance of the theme and the negative impact of experiencing infertility, in order to seek means of justice in the equal treatment of patients.

### Electronic supplementary material

Below is the link to the electronic supplementary material.

Supplementary Material 1

## Data Availability

The datasets used and/or analysed during the current study are available from the corresponding author on reasonable request.
