# Supplementary material for: The search for the principle of justice for infertile couples: characterization of the brazilian population and bioethical discussion
Source: BMC Med Ethics. 2023 Sep 4;24:69. doi: 10.1186/s12910-023-00947-4 (PMC10476313; doi:10.1186/s12910-023-00947-4)
Supplement: Supplementary file 1 — Supplementary Material 1 [file 12910_2023_947_MOESM1_ESM.pdf]

Supplementary file 1.

The search for the principle of justice for infertile couples: Characterization of the Brazilian population and bioethical discussion.

For each question, please select the answer that best reflects how you think and feel.

1. **How old are both partners in the couple?**

2. **What is your education level?**
   Elementary school [ ]
   High school [ ]
   College degree [ ]
   Incomplete college education [ ]
   Post-graduation [ ]
   None of the alternatives [ ]

3. **What is your partner's education level?**
   Elementary school [ ]
   High school [ ]
   College degree [ ]
   Incomplete college education [ ]
   Post-graduation [ ]
   None of the alternatives [ ]

4. **What is your color or race?**
   White [ ]
   Black [ ]
   Asian [ ]
   Brown [ ]
   Indigenous [ ]

5. **What is your partner's color or race?**
   White [ ]
   Black [ ]
   Asian [ ]
   Brown [ ]
   Indigenous [ ]

6. **What is the city where the couple resides?**

7. **What is the city where the couple will undergo infertility treatment?**

8. **What is the duration of infertility?**

9. **What was the number of attempts (treatment) to get pregnant?**

10. What is the cause of infertility?

Female [ ]
Male [ ]
Mixed factor [ ]
Infertility without apparent cause [ ]

11. Does the couple already have children?

Yes [ ]
No [ ]

12. How does/did infertility impact the couple's life?

Strengthened the relationship [ ]
Weakened the relationship [ ]
No positively or negatively impact [ ]

13. Did infertility and the search for treatment cause moments of anxiety/stress in the couple's life?

Yes [ ]
Yes, only after some treatment attempts [ ]
No [ ]

14. The couple considers their quality of life:

Excellent [ ]
Good [ ]
Bad [ ]

15. Access to specialist and medical services to treat infertility was:

Easy [ ]
Easy, only after indication/help [ ]
Difficult [ ]

16. Access to the assisted reproduction clinic and infertility treatment options was:

Easy [ ]
Easy, after searching in another city/state [ ]
Difficult [ ]

17. Has the couple ever thought about giving up on infertility treatment due to some access difficulty?

Yes [ ]
No [ ]

18. Did the couple feel welcomed in seeking medical services and/or treatment for infertility?

Yes [ ]
Yes, after some frustrations [ ]
No [ ]

19. Did the couple seek more than one medical service to treat infertility?

Yes, searched for the best welcome service [ ]
Yes, searched for adequate service financially [ ]
No, because there are not many options in your location [ ]
No, as it does not know other possibilities [ ]

20. Has the couple sought or is aware of free treatment in other countries?

Yes. Has knowledge, but has not tried treatment [ ]
Yes. Has knowledge, but has not obtained access to the other country [ ]
No, no knowledge [ ]

21. Point out the greatest difficulty encountered by the couple to access medical services and/or treatment for infertility:

Location [ ]
Financial [ ]
Location + Financial [ ]
Discrimination [ ]
Other [ ]
Did not find any difficulty in access [ ]

22. Has the cost of treatment impacted the family budget?

Yes [ ]
No [ ]

23. Was there a need to take out loans, have assets or a similar situation to pay for infertility treatment?

Yes [ ]
No [ ]

24. Was there a need to wait between the diagnosis, the indication of treatment and the performance of the procedure to obtain financial resources?

Yes [ ]
No [ ]

25. Did the couple seek coverage from health care providers or the public health system for treatment of infertility?

Yes, but they did not find any [ ]
No [ ]

26. The couple considers their socioeconomic status:

Excellent [ ]
Good [ ]
Bad [ ]

27. What is the gross household income?

R$ 3.000 a R$ 5.000 [ ]
R$ 5.000 a R$ 10.000 [ ]
R$ 10.000 a R$ 15.000 [ ]
R$ 15.000 a R$ 20.000 [ ]
R$ 25.000 or more [ ]
None of the alternatives [ ]

28. Does the couple have friends seeking treatment for infertility?

Yes [ ]
No [ ]

29. If you know, did friends get access to infertility treatment?

Yes [ ]
Yes, with difficulty [ ]
No [ ]

30. If desired, enter a comment.
